# Three-dimensional conditional generative adversarial network-based virtual thin-slice technique for the morphological evaluation of the spine

**DOI:** 10.1038/s41598-022-16637-x

**Published:** 2022-07-16

**Authors:** Atsushi Nakamoto, Masatoshi Hori, Hiromitsu Onishi, Takashi Ota, Hideyuki Fukui, Kazuya Ogawa, Jun Masumoto, Akira Kudo, Yoshiro Kitamura, Shoji Kido, Noriyuki Tomiyama

**Affiliations:** 1 Department of Diagnostic and Interventional Radiology, Osaka University Graduate School of Medicine, 2-2, Yamadaoka, Suita, Osaka 565-0871 Japan; 2 Department of Radiology, Kobe University Graduate School of Medicine, 7-5-2, Kusunoki-cho, Chuo-ku, Kobe, Hyogo 650-0017 Japan; 3 Medical System Research and Development Center, FUJIFILM Corporation, 26-30, Nishiazabu 2-Chome, Minato-ku, Tokyo, 106-8620 Japan; 4 Imaging Technology Center, FUJIFILM Corporation, 26-30, Nishiazabu 2-Chome, Minato-ku, Tokyo, 106-8620 Japan; 5 Department of Artificial Intelligence Diagnostic Radiology, Osaka University Graduate School of Medicine, 2-2, Yamadaoka, Suita, Osaka 565-0871 Japan

**Keywords:** Musculoskeletal system, Trauma

## Abstract

Virtual thin-slice (VTS) technique is a generative adversarial network-based algorithm that can generate virtual 1-mm-thick CT images from images of 3–10-mm thickness. We evaluated the performance of VTS technique for assessment of the spine. VTS was applied to 4-mm-thick CT images of 73 patients, and the visibility of intervertebral spaces was evaluated on the 4-mm-thick and VTS images. The heights of vertebrae measured on sagittal images reconstructed from the 4-mm-thick images and VTS images were compared with those measured on images reconstructed from 1-mm-thick images. Diagnostic performance for the detection of compression fractures was also compared. The intervertebral spaces were significantly more visible on the VTS images than on the 4-mm-thick images (*P* < 0.001). The absolute value of the measured difference in mean vertebral height between the VTS and 1-mm-thick images was smaller than that between the 4-mm-thick and 1-mm-thick images (*P* < 0.01–0.54). The diagnostic performance of the VTS images for detecting compression fracture was significantly lower than that of the 4-mm-thick images for one reader (*P* = 0.02). VTS technique enabled the identification of each vertebral body, and enabled accurate measurement of vertebral height. However, this technique is not suitable for diagnosing compression fractures.

## Introduction

Multidetector-row CT (MDCT) is currently in widespread use worldwide, and can reconstruct thin-slice images with a slice thickness of 1 mm or less. Thin-slice images are useful for evaluating small lesions and depicting detailed structures, and are also used for reconstructing multiplanar reformatted images and 3-dimensional (3D) images using processes such as volume rendering and maximum intensity projection^1^. Sagittal reformatted images are useful for evaluating spinal lesions and morphology^2,3^, for numbering the vertebrae, and identifying the vertebral level of lesions. However, it is not possible to reconstruct thin-slice images after the raw data have been deleted from storage in the scanner. Although saving thin-slice images for all patients before deleting the raw data would be one way of solving this problem, it would require more storage in picture archiving and communication system (PACS), which is costly.

Artificial intelligence (AI) has been attracting greater attention in recent years and many reports regarding the use of AI in radiological image analysis have been published^4–7^. Generative adversarial networks (GANs) are deep learning models that comprise two networks trained simultaneously: a generator and a discriminator, and are aimed at generating new images^8^. GANs are being increasingly employed for various radiology applications, such as in noise reduction of CT images and for generating images of different modalities^8–13^. Virtual thin-slice (VTS) technique is a newly developed GAN-based algorithm that can generate 1-mm-thick virtual images from CT images obtained with a slice thickness of 3–10 mm^14^. This software is now commercially available and can be applied to CT images obtained with any CT scanner. If VTS images are as useful as true thin-slice images, then thin-slice images would not need to be prepared in advance and could be created when needed. It would also make it possible to create thin-slice images for comparison, even when thin-slice images have not been saved in previous examinations. However, it is necessary to verify the diagnostic ability of VTS images before this technique can be applied to clinical practice. Although many studies have reported use of the GAN technique for creating medical images^8,12,13^, few have examined whether the created images have the same diagnostic ability as the true images. The purpose of this study was to investigate whether VTS images can be used as a substitute for true thin-slice images in evaluation of the spine by analyzing the visibility of the vertebral bodies, accuracy of vertebral body height measurement, and diagnostic performance in detecting compression fractures.

## Materials and methods

This retrospective study was approved by the Osaka University Clinical Research Review Committee, and the requirement for informed consent was waived by the Osaka University Clinical Research Review Committee. All methods were carried out in accordance with relevant guidelines and regulations. Patients who underwent CT for evaluation of aortic or cardiac disease were eligible for inclusion in this study because we obtained a single scan in one breath-hold from the supraclavicular area to the symphysis pubis in these patients, whereas separate scans were obtained for the chest and abdominopelvic regions in other patients. Enrolled were 73 consecutive patients who underwent CT between January and February 2019 or between December 2020 and January 2021 (50 men and 23 women; age range, 25–91 years; mean age, 72.9 years). The clinical indications for CT in these patients are listed in Table 1.

**Table 1:** Clinical indications for CT in the enrolled patients.

Follow up after treatment for TAA and/or AAA	27
Preoperative evaluation of cardiac disease	18
Preoperative evaluation of TAA or AAA	7
Follow up after treatment for DAA	5
Follow up for untreated DAA	5
Follow up after cardiac surgery	4
Follow up after TAVI	3
Follow up for untreated TAA	1
Follow up for untreated EIAA	1
Evaluation of aortitis	1
Acute back pain	1

*TAA* thoracic aortic aneurysm, *AAA* abdominal aortic aneurysm, *DAA* dissecting aortic aneurysm, *EIAA* external iliac arterial aneurism, *TAVI* transcatheter aortic valve implantation.

### CT examination

CT was performed using a 160- or 320-slice CT scanner (Aquilion Precision, Canon Medical Systems, Otawara, Japan, n = 34, or Aquilion ONE GENESIS Edition, Canon Medical Systems, n = 39). A pre-contrast scan was performed in all patients from the supraclavicular area to the symphysis pubis during a single breath hold. Tube current was adjusted individually using an auto-exposure control technique with a standard deviation setting of 15. The remaining scan parameters were as follows: tube voltage, 120 kVp; rotation time, 0.5 s; helical pitch, 0.83. Although post-contrast scans were also acquired in 31 patients, only the pre-contrast images were used in this study.

From the raw data of each patient, two sets of axial images were reconstructed, with a slice thickness/interval of 4/4 and 1/1 mm. A hybrid iterative reconstruction algorithm (AIDR 3D, Canon Medical Systems) with a weak strength setting was applied. The remaining reconstruction parameters were as follows: kernel, FC03; reconstruction field of view, 350 mm (pixel size, 0.68 × 0.68 mm).

### Virtual thin-slice technique

**Figure 1:**
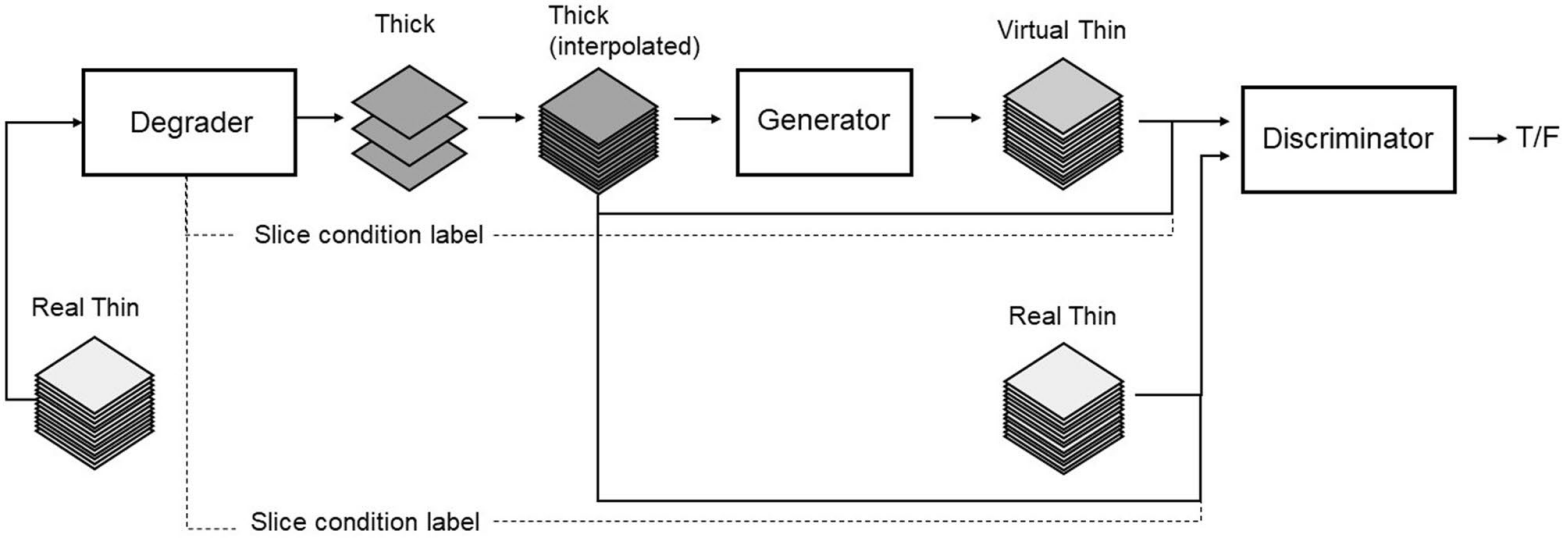
Adversarial training framework for thick–thin slice translation of CT images.

VTS is a conditional-GAN based algorithm. Thick-slice images with slice thickness/intervals of 3–10 mm were randomly simulated from real thin-slice images by down-sampling with Gaussian smoothing. A pair of original thin-slice images and simulated thick-slice images were used to train the VTS generator in the GAN framework (Fig. 1). The generator is an encoder-decoder type architecture with skip connections inspired by U-Net to reconstruct high resolution images. The role of the discriminator is to enable the generator to output virtual thin-slice images that are hard to distinguish from real ones. Both the generator and the discriminator are composed of 3D Convolutional Neural Networks. The conditioning labels (e.g. slice interval) associated with input thick images are fed into the discriminator to improve the accuracies of super resolution. While generator training, L1 loss was calculated in addition to adversarial loss, to minimize the pixel-wise intensity difference between the original (ground truth) and the generated thin-slice images, as these should be as close as possible. VTS software is a function of the PACS viewer (SYNAPSE SAI Viewer Version 1.0, FUJIFILM, Tokyo, Japan), which has regulatory approval in Japan. The training CT data for this software contained CT images of various body parts (head, chest, abdomen, and legs) obtained with scanners of various manufacturers. Thus, the software can be applied to any part of the body. The generated VTS images were isotropic with voxel size of 1 × 1 × 1 mm. The details of the VTS technique have been presented at a previous conference, and the manuscript is available for reference on the preprint server^14^. VTS software was applied to the 4-mm-thick data set of each patient to generate 1-mm-thick VTS images.

### Qualitative analysis

Two radiologists familiar with abdominal radiology (9 and 6 years’ experience) independently reviewed the sagittal images reformatted from 4-mm-thick images and the VTS images and evaluated the visibility of the intervertebral spaces in each of four regions: cervical, upper thoracic, lower thoracic, and lumbar spine. They reviewed these images on a commercially available workstation (SYNAPSE VINCENT version 5.3.001, FUJIFILM), and assigned a score using the following 4-point scale: 4, all intervertebral spaces are visible; 3, most intervertebral spaces are visible but some are unclear; 2, most intervertebral spaces are unclear; 1, no intervertebral spaces are visible. The radiologists were informed that the images for evaluation were either 4-mm-thick or VTS images, but were blinded to the patients’ identity, medical background, and the reconstruction protocol used.

### Quantitative analysis

Two radiologists familiar with abdominal radiology (16 and 9 years’ experience), different to the radiologists who performed the qualitative assessment, independently measured the height of the first thoracic (Th1) and first lumbar (L1) vertebrae on sagittal reformatted images made from each of the 4-mm-thick, true 1-mm-thick, and VTS data sets. Height was measured at the anterior border of each of these vertebrae. The absolute values of the difference between the measured heights on the 4-mm-thick and true 1-mm-thick images (D_1_) were calculated, as well as the absolute values of the difference between the measured heights on VTS and true 1-mm-thick images (D_2_). The absolute percentage errors between the measured heights on the 4-mm-thick and true 1-mm-thick images (%Error_1_) was also calculated by dividing D_1_ by the measured height on true 1-mm-thick images, as well as the absolute percentage errors between the measured heights on VTS and true 1-mm-thick images (%Error_2_). Measurements were performed using a workstation (SYNAPSE VINCENT version 5.3.001).

### Diagnostic performance in detecting compression fracture

The same two radiologists who performed the qualitative assessment also independently evaluated the possible presence of compression fracture using the sagittal reformatted images constructed from each of the 4-mm-thick images and the VTS images. They classified the likelihood of compression fracture in all vertebrae using the following 4-point confidence score scale: 1, probably no fracture present; 2, indefinite presence of fracture; 3, fracture probably present; and 4, fracture definitely present. Before the assessment, they were informed that a confidence level of 3 or 4 would be considered a positive finding for the calculation of sensitivity and positive predictive value (PPV). The criteria for compression fracture used in this study were: 1, ratio of the anterior height of the vertebra (AH) to the posterior height (PH) < 0.75; 2, ratio of the central height of the vertebrae (CH) to AH or PH < 0.8; 3, height of a vertebra reduced by > 20% compared with those above and below^15^. The reference standard was determined by two other radiologists (16 and 9 years’ experience) who evaluated the presence or absence of compression fracture on sagittal images reformatted from the true 1-mm-thick images, in consensus.

### Statistical analysis

Visual scores regarding the visibility of intervertebral spaces were compared using Wilcoxon signed rank test. The absolute values of the difference in measured vertebral heights (D_1_ and D_2_) were compared using paired *t*-test. The absolute percentage errors of the measured vertebral heights (%Error_1_ and %Error_2_) were also compared using paired *t*-test. Interobserver agreement for each of D_1_ and D_2_ was evaluated by intraclass correlation coefficient (ICC). To analyze diagnostic performance for detecting compression fracture, jackknife free-response receiver-operating characteristic (JAFROC) analysis was performed using JAFROC software (JAFROC Version 4.2.1, www.devchakraborty.com). This software computes the figure of merit (FOM), which is defined as the probability that a lesion is rated higher than the highest rated non-lesion on a normal image^16^. In the present study, JAFROC1 was used rather than JAFROC or JAFROC2 because of its high statistical power for human observers^17^. For all tests, a *P* value less than 0.05 was considered significant.

## Results

### Qualitative analysis

**Figure 2:**
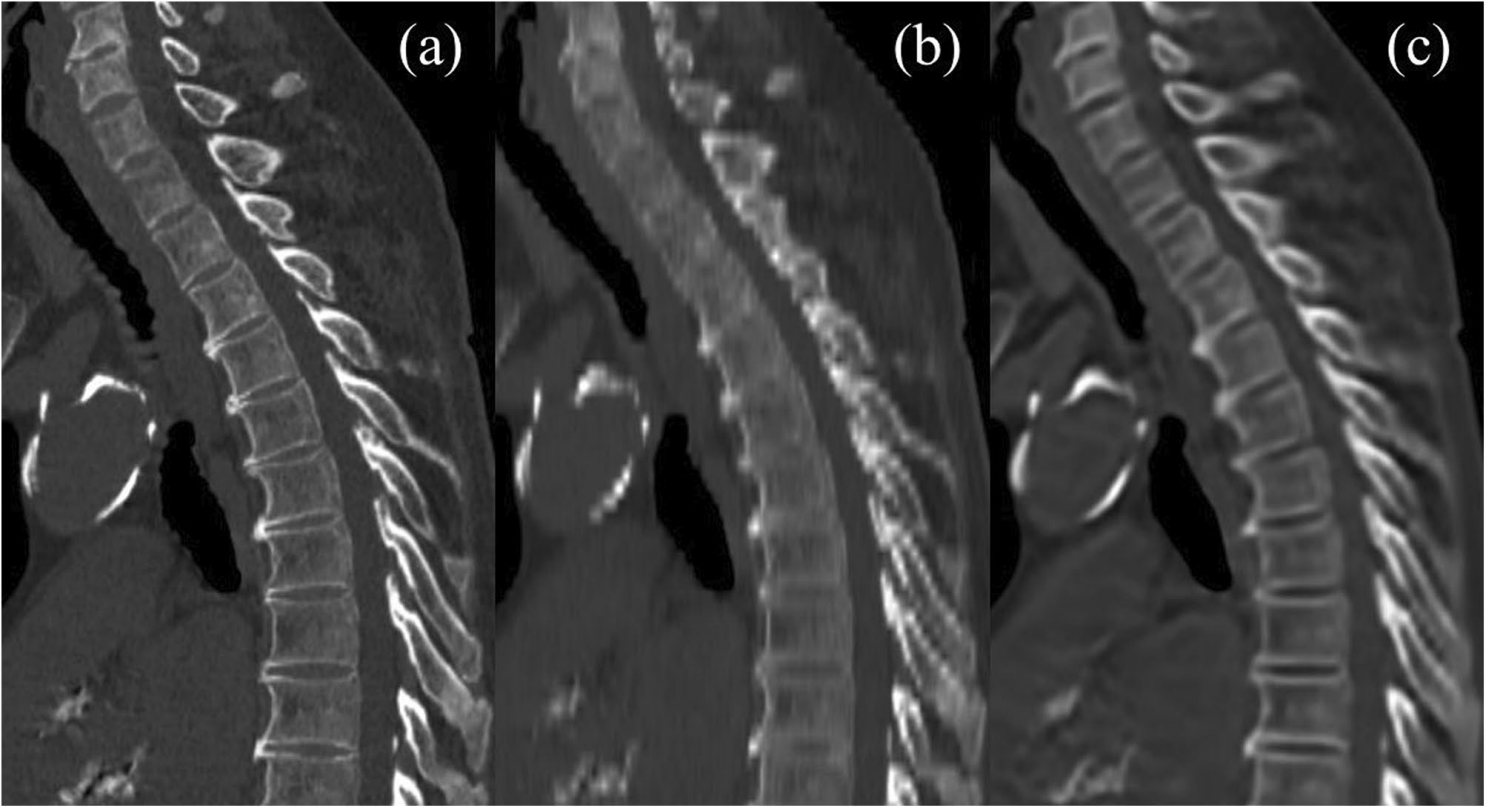
Sagittal reformatted images reconstructed from 1-mm-thick images (**a**), 4-mm-thick images (**b**), and virtual thin-slice images (**c**). The intervertebral spaces of the cervical and upper thoracic spine are not clearly depicted on the reconstruction from 4-mm-thick images. On the reconstruction of virtual thin-slice images, the intervertebral spaces are more clearly depicted and it is easier to recognize the shapes of the vertebral bodies.

Mean visual scores regarding the visibility of intervertebral spaces are summarized in Table 2. The mean score was significantly higher for VTS than for 4-mm-thick images for all regions for both readers (*P* < 0.001) (Fig. 2).

**Table 2:** Mean scores of visibility of intervertebral spaces.

	Reader 1			Reader 2		
	Thick slice (4 mm)	VTS	*P* value	Thick slice (4 mm)	VTS	*P* value
Cervical spine	2.0 ± 0.5	2.5 ± 0.7	< 0.001	1.2 ± 0.5	2.5 ± 0.8	< 0.001
Upper thoracic spine	2.0 ± 0.3	2.5 ± 0.6	< 0.001	1.0 ± 0.2	2.6 ± 0.7	< 0.001
Lower thoracic spine	2.7 ± 0.5	3.2 ± 0.6	< 0.001	1.7 ± 0.6	3.4 ± 0.6	< 0.001
Lumbar spine	3.5 ± 0.5	3.7 ± 0.5	< 0.001	3.1 ± 0.7	4.0 ± 0.2	< 0.001

Data are mean score (rated on a 4-point scale) ± standard deviation.

*VTS* virtual thin slice.

### Quantitative analysis

The mean measured heights of Th1 and L1 are summarized in Table 3. The mean absolute value of the difference in measured height between VTS and true 1-mm-thick images (D_2_) was less than that between 4-mm-thick and true 1-mm-thick images (D_1_) for both readers, and the difference was significant for L1 measured by Reader 1 (*P* < 0.01). The mean absolute percentage error of the measured height between VTS and true 1-mm-thick images (%Error_2_) was smaller than that between 4-mm-thick and true 1-mm-thick images (%Error_1_) for both readers, and the difference was significant for L1 measured by Reader 1 (*P* < 0.01). The ICCs of the two readers for height measured on the 4-mm-thick images were 0.461 and 0.795 for Th1 and L1, respectively, whereas those measured on VTS were 0.524 and 0.813 for Th1 and L1, respectively.

**Table 3:** Measured heights of thoracic and lumbar vertebrae.

	Thick slice (4 mm)	VTS	Thin slice (1 mm)	D_1_	D_2_	*P* value	%Error_1_	%Error_2_	*P* value
**Reader 1**									
Th1	14.0 ± 1.9	14.0 ± 1.7	15.2 ± 1.6	1.5 ± 1.2	1.4 ± 0.9	0.30	9.9 ± 7.7%	9.0 ± 6.0%	0.33
L1	23.4 ± 3.4	22.9 ± 3.2	23.1 ± 3.2	1.2 ± 1.4	0.9 ± 1.2	< 0.01	5.6 ± 7.6%	4.3 ± 6.6%	< 0.01
**Reader 2**									
Th1	13.1 ± 2.3	13.1 ± 2.1	13.8 ± 1.6	1.6 ± 1.6	1.5 ± 1.2	0.54	12.0 ± 11.9%	11.1 ± 9.4%	0.53
L1	22.5 ± 3.5	22.1 ± 3.2	22.5 ± 3.0	1.5 ± 1.1	1.3 ± 1.1	0.22	6.9 ± 5.3%	5.8 ± 4.9%	0.16

Data are mean ± standard deviation (mm, except for %Error_1_ and %Error_2_).

*VTS* virtual thin slice, *Th1* first thoracic vertebra, *L1* first lumber vertebra, *D*_*1*_ absolute value of the difference between measured heights on thick slice images and those on thin slice images, *D*_*2*_ absolute value of the difference between measured heights on VTS images and those on thin slice images, *%Error*_*1*_ absolute percentage error between measured heights on thick slice images and those on thin slice images, *%Error*_*2*_ absolute percentage error between measured heights on VTS images and those on thin slice images.

### Diagnostic performance in detecting compression fracture

**Figure 3:**
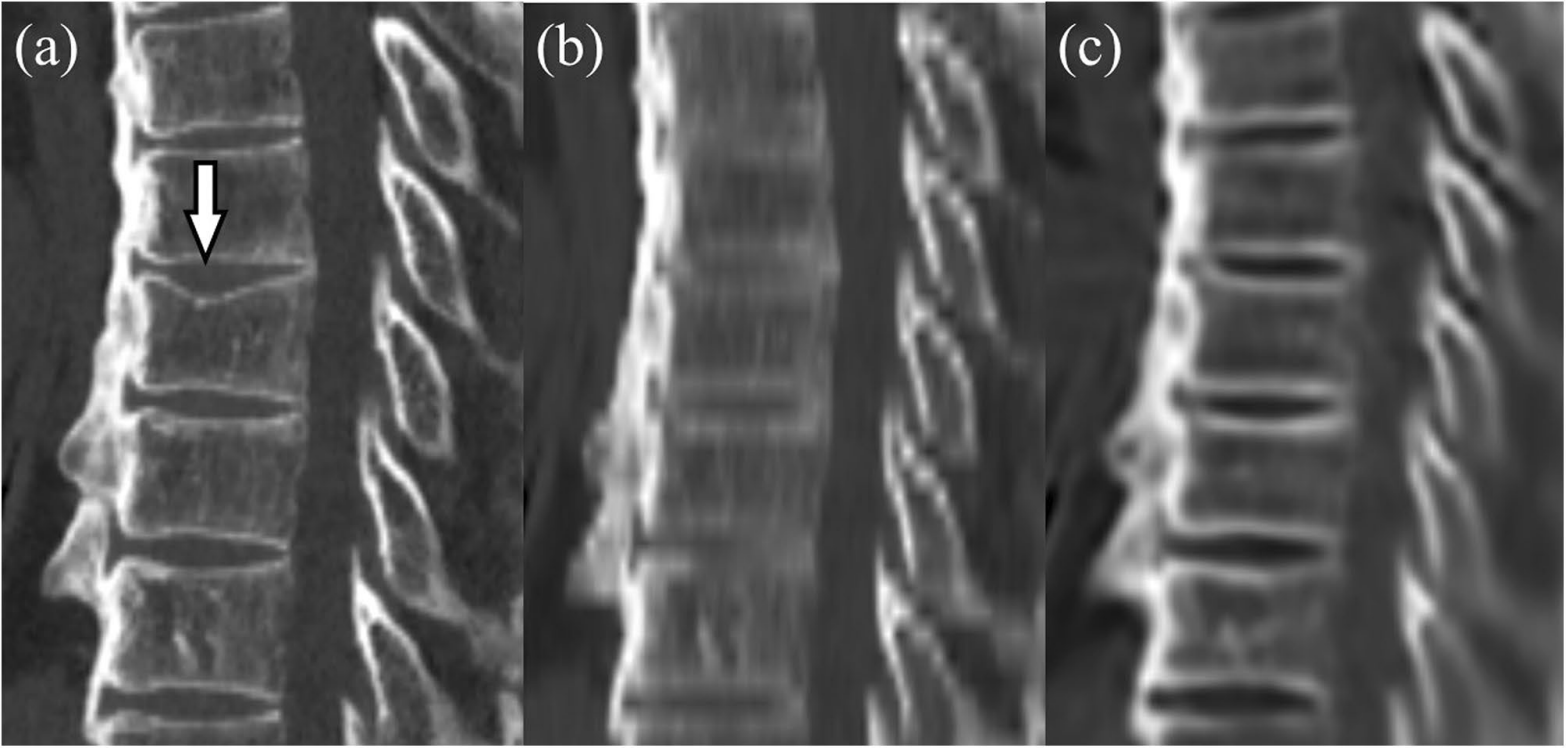
Sagittal reformatted images reconstructed from 1-mm-thick images (**a**), 4-mm-thick images (**b**), and virtual thin-slice images (**c**). A compression fracture of the 8th thoracic vertebra is seen on the reconstruction from 1-mm-thick images (arrow), but is not depicted on that from virtual thin-slice images.

**Figure 4:**
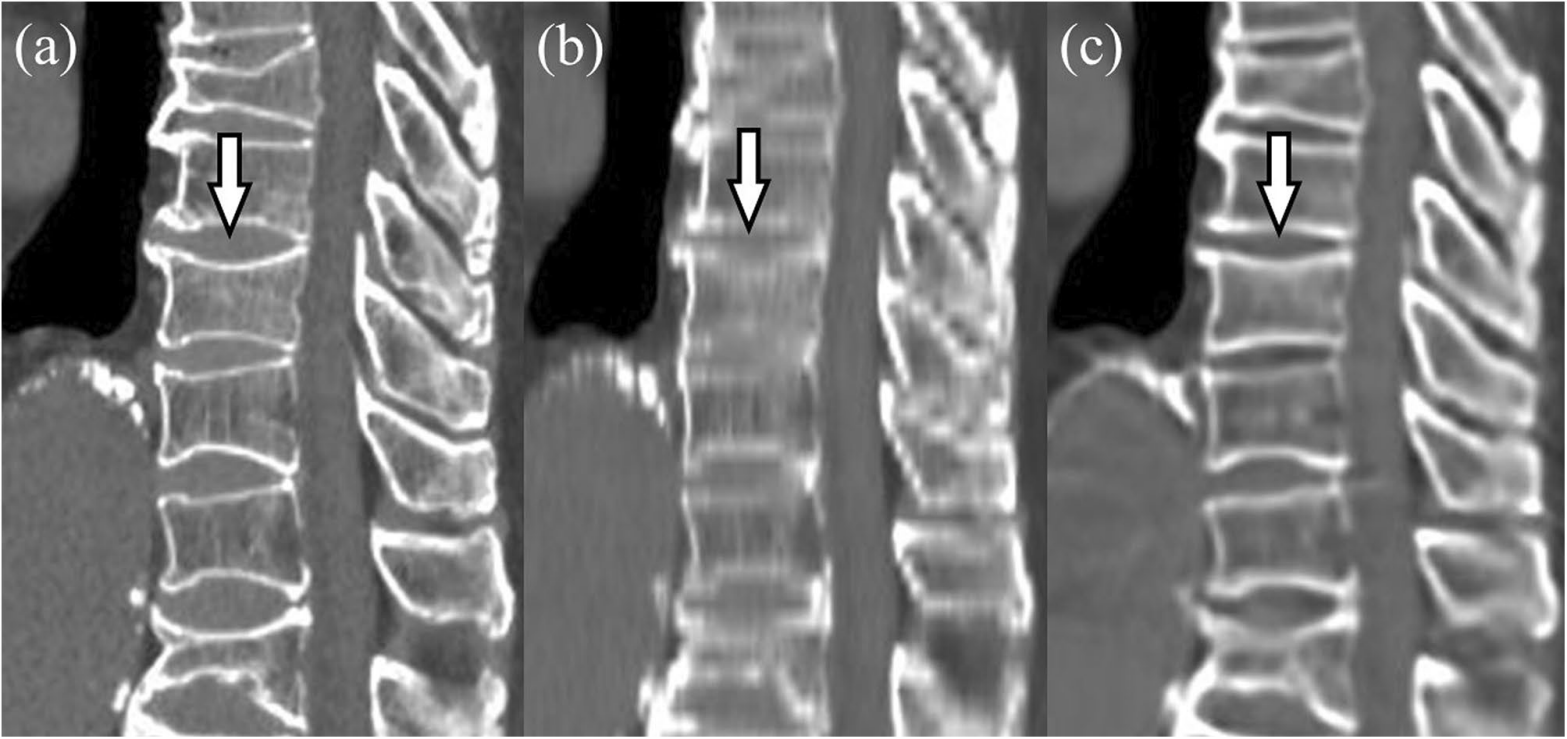
Sagittal reformatted images reconstructed from 1-mm-thick images (**a**), 4-mm-thick images (**b**), and virtual thin-slice images (**c**). Multiple compression fractures are seen in the thoracic and lumbar spine. The fracture of the 10th thoracic vertebra can be seen on the reconstruction from 1-mm-thick images (arrow), and is also identifiable on that from the 4-mm-thick images. However, it is barely visible on that from the virtual thin-slice image, and is therefore difficult to diagnose.

**Figure 5:**
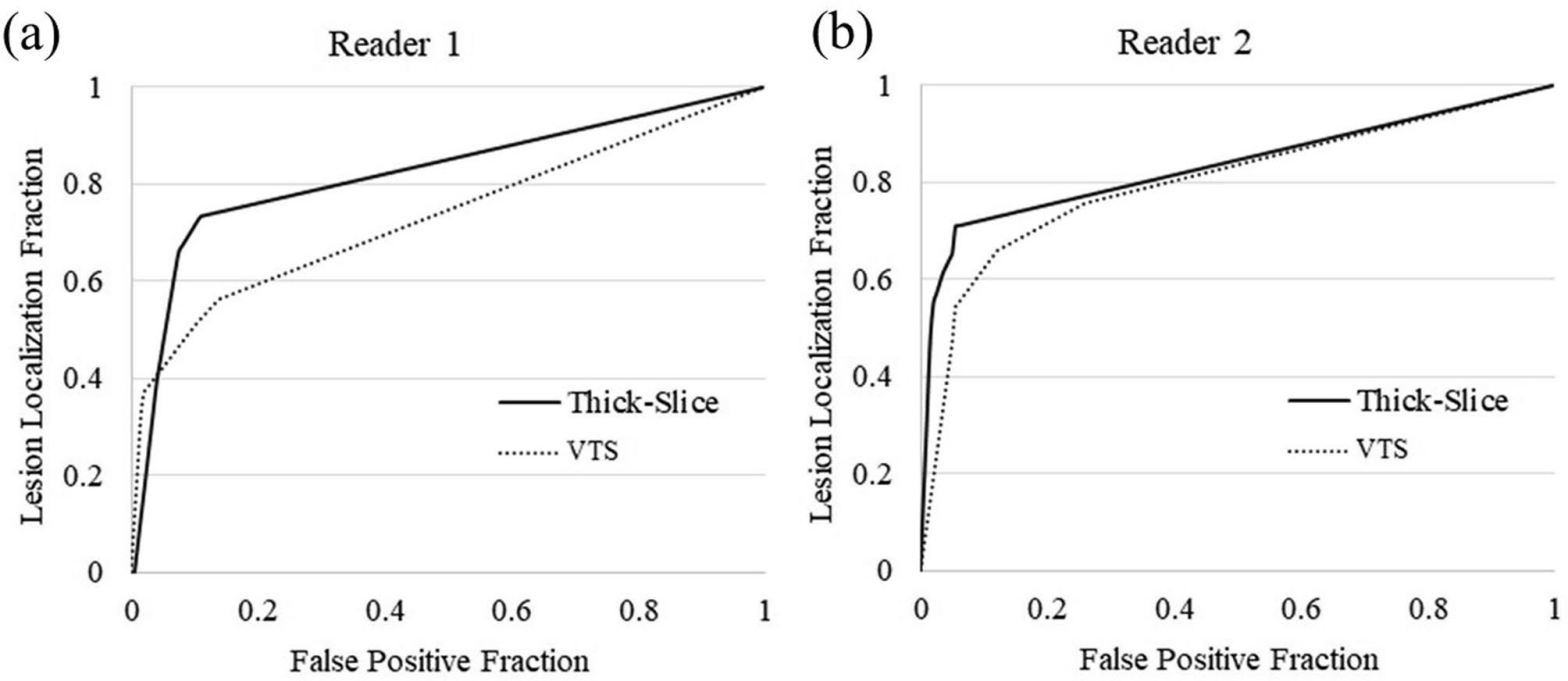
JAFROC curves for Reader 1 (**a**) and Reader 2 (**b**). The figure of merit was significantly higher for thick-slice images than for virtual thin-slice images for Reader 1 (*P* = 0.02).

A compression fracture was detected in 41 vertebrae in 15 patients by consensus reading of the true 1-mm-thick images. Diagnostic performance for detecting compression fractures is summarized in Table 4. Some compression fractures that were correctly diagnosed by both readers on 4-mm-thick images were missed on VTS (Figs. 3, 4). Sensitivity, positive predictive value, and FOM were lower for the VTS images than for the 4-mm-thick images in both readers, and the difference was statistically significant for FOM for Reader 1 (*P* = 0.02) (Fig. 5).

**Table 4:** Diagnostic performance for detecting compression fractures.

	Reader 1			Reader 2		
	Thick slice (4 mm)	VTS	*P* value	Thick slice (4 mm)	VTS	*P* value
Sensitivity	0.66 (27/41)	0.51 (21/41)	0.26	0.61 (25/41)	0.54 (22/41)	0.51
Positive predictive value	0.75 (27/36)	0.66 (21/32)	0.43	0.89 (25/28)	0.76 (22/29)	0.30
JAFROC1 figure of merit	0.82	0.73	0.02	0.83	0.80	0.66

Numbers in parentheses are actual numbers of lesions.

*VTS* virtual thin slice, *JAFROC* Jackknife alternative free-response receiver-operating characteristic.

## Discussion

GAN is a type of deep learning model capable of generating realistic-looking fake images^8^. In recent years, GANs have been used for various radiology applications, such as in noise reduction of CT images^9–11^, augmentation of data for deep learning algorithm training^18^, and for generating images of different modalities^12,13^. VTS is a newly developed GAN-based algorithm that can generate virtual images of 1-mm thickness from thick-slice CT images of 3–10-mm thickness. Although VTS can be applied to any part of the body, it is considered to be more effective in high-contrast regions such as bone^14^. Thus, we conducted the present study to investigate the utility of VTS for morphological evaluation of the spine. The results of our qualitative analysis demonstrated that the visibility of intervertebral spaces was higher on sagittal reformatted images created from VTS images than on reformatted images made from 4-mm-thick images, for all spinal regions. The intervertebral spaces of the cervical and upper thoracic spine were hardly visible on reformatted images made from the 4-mm-thick images (mean score, 1.0–2.0), but visibility was improved on reformatted images made from VTS images (mean score, 2.5–2.6). Thus, reformatted images made from VTS images would make it easier to recognize and number individual vertebral bodies, and thus identify the vertebral level of a lesion. Moreover, this technique would improve the quality of reformatted or 3D images, and it would make it easier to obtain an overview of the whole spine.

The absolute values of the differences in measured heights of thoracic and lumbar vertebrae between VTS and true 1-mm-thick images were smaller than those between 4-mm-thick and true 1-mm-thick images, and interobserver agreement was slightly improved using VTS compared with 4-mm-thick images. Therefore, reformatted VTS images might have the potential to achieve more accurate measurement of bone structures compared with reformatted images using 4-mm-thick images. However, the height tends to be underestimated when using VTS, and it remains unclear whether VTS can be used for quantitative evaluation instead of thin-slice images. Further studies will be necessary to confirm the usefulness of VTS images in quantitative evaluation.

Regarding diagnostic ability for compression fracture, VTS had impaired performance compared with 4-mm-thick images, for the reason that slight compression fractures were sometimes not depicted correctly by VTS images (Fig. 3). If localized mild depressions suggestive of a mild compression fracture are not visible on the 4-mm-thick images, then the VTS images generated from these images are also unlikely to contain such information. Moreover, some compression fractures were depicted less definitively on the VTS images than the 4-mm-thick images (Fig. 4). If a mild compression fracture is located near the boundary between two adjacent 4-mm-thick images, it might be recognizable on the reformatted 4-mm-thick images. However, in the process of VTS generation, there might be a tendency to make the morphology of vertebral bodies closer to normal vertebrae, which might obscure such a slight compression fracture. This might be the reason for the impaired diagnostic performance. VTS was originally developed for purposes such as improving the visibility of the vertebral bodies, and not for the diagnosis of lesions such as compression fractures. Thus, as indicated by the present results, the current VTS technique would not be suitable for the evaluation of subtle abnormalities. Although training using more cases, including those with compression fractures, might improve the diagnostic ability of VTS, it is unclear whether it is really possible for the trained algorithm to accurately delineate subtle lesions. Further improvement and validation will be necessary before VTS can be used for the purpose of diagnosing lesions. Our results would suggest that virtual images generated by a GAN would not always accurately depict pathological abnormalities, and this might be also true for other types of GANs, such as noise reduction, super-resolution, and generating images of different modalities. Thus, these virtual images would need to be validated before use for diagnostic purposes in routine clinical practice.

Our study had several limitations. First, this was a retrospective study, and the number of patients was relatively small. Second, although thick-slice images of 3–10-mm thickness can be applied to VTS software, we evaluated only 4-mm-thick images. Because thick-slice images with thickness 4 or 8 times that of the thin-slice images were used when training the VTS^14^, it was considered that images with a thickness of 4 mm were the most suitable for this software. Third, the reference standard for compression fractures was determined by consensus reading of the true 1-mm-thick CT images, and other diagnostic modalities such as MR imaging were not performed. As the purpose of this study was to investigate whether VTS images could be used as a substitute for true thin-slice images, it was appropriate to use the thin-slice images as the gold standard.

## Conclusions

Virtual thin-slice technique enabled the identification of all vertebral bodies and more accurate measurement of vertebral height compared with thick-slice images, but is not suitable for the detection of compression fractures. Further improvements are needed before virtual thin-slice images can achieve the same diagnostic performance as true thin-slice images for detecting lesions.

## Data Availability

The datasets generated during and/or analyzed during the current study are available from the corresponding author on reasonable request.
